# Fractional Flow Reserve Relates Stronger to Coronary Plaque Burden Than Nonhyperemic Pressure Indexes

**DOI:** 10.1161/JAHA.124.039324

**Published:** 2025-02-19

**Authors:** Ruurt A. Jukema, Jorge Dahdal, Nick S. Nurmohamed, Pieter G. Raijmakers, Jos Twisk, Pepijn A. van Diemen, R. Nils Planken, G. Aernout Somsen, Niels J. Verouden, Guus A. de Waard, Paul Knaapen, Ibrahim Danad, Roel Driessen

**Affiliations:** ^1^ Department of Cardiology Amsterdam Cardiovascular Sciences, Amsterdam University Medical Center, Vrije Universiteit Amsterdam Amsterdam the Netherlands; ^2^ Department of Medicine Hospital Del Salvador Santiago Chile; ^3^ Division of Cardiology The George Washington University School of Medicine Washington DC; ^4^ Department of Radiology and Nuclear Medicine Amsterdam University Medical Center, Vrije Universiteit Amsterdam Amsterdam the Netherlands; ^5^ Epidemiology and Data Science Amsterdam University Medical Center, Vrije Universiteit Amsterdam Amsterdam the Netherlands; ^6^ Department of Radiology and Nuclear Medicine, Amsterdam Cardiovascular Sciences Amsterdam University Medical Center location UvA Amsterdam the Netherlands; ^7^ Cardiology Centers of the Netherlands Amsterdam the Netherlands; ^8^ Department of Cardiology Radboud University Medical Center Nijmegen the Netherlands; ^9^ Department of Cardiology, Division of Heart and Lungs Utrecht University, Utrecht University Medical Center Utrecht the Netherlands

**Keywords:** computed coronary tomography angiography, fractional flow reserve, high‐risk plaque, instantaneous wave‐free ratio, percentage atheroma volume, resting distal pressure/arterial pressure, resting full‐cycle ratio, Angiography, Computerized Tomography (CT)

## Abstract

**Background:**

The relationship between fractional flow reserve (FFR), resting full‐cycle ratio (RFR), instantaneous wave‐free ratio (iFR), resting distal pressure/aortic pressure (Pd/Pa), and plaque burden as well as phenotype requires further elucidation.

**Methods and Results:**

In this single‐center cohort study, patients with suspected coronary artery disease who underwent invasive coronary angiography, including routine hyperemic (FFR) and nonhyperemic invasive pressure (Pd/Pa and iFR or RFR) interrogation and computed coronary tomography angiography were prospectively enrolled. Computed coronary tomography angiography was used to assess percentage atheroma volume (PAV), positive remodeling, and low‐attenuation plaque.

Among 241 patients with 556 vessels, FFR correlated stronger to PAV compared with Pd/Pa (r=−0.56; versus r=−0.43; *P*<0.01) and iFR/RFR (r=−0.47; *P*=0.04). Vessels with FFR and Pd/Pa discordancy showed higher PAV in case of abnormal FFR (34% versus 14%; *P*<0.01), whereas vessels with FFR and iFR/RFR discordancy showed similar PAV levels. FFR and iFR/RFR, but not Pd/Pa, were independently associated with the presence of low‐attenuation plaque (β, −0.03, *P*<0.01; β, −0.03, *P*=0.01; and β, −0.02, *P*=0.10, respectively). None of the invasive pressure measurements was independently associated with positive remodeling. Pressure index discordancy was not associated with positive remodeling or low‐attenuation plaque.

**Conclusions:**

FFR correlated stronger to plaque burden, as defined by PAV, than nonhyperemic pressure indexes. For plaque phenotype, both FFR and iFR/RFR were independently associated with low‐attenuation plaque, whereas none of the invasive pressure indexes was associated with positive remodeling.

**Nonstandard Abbreviations and Acronyms**
AI‐QCT: artificial intelligence‐guide quantitative coronary computed tomography angiography analysis
DS: diameter stenosis
FFR: fractional flow reserve
ICA: invasive coronary angiography
iFR: instantaneous wave‐free ratio
LAP: low‐attenuation plaque
PAV: percentage atheroma volume
Pd/Pa: resting distal pressure/arterial pressure
PR: positive remodeling
RFR: resting full‐cycle ratio

**Clinical Perspective**

**What Is New?**

- Our findings suggest that fractional flow reserve provides a superior assessment of plaque burden compared with nonhyperemic pressure indexes.
- Both fractional flow reserve and instantaneous wave‐free ratio/resting full‐cycle ratio, but not resting distal pressure/arterial pressure, were associated with the presence of low‐attenuation plaque, whereas none of the invasive pressure indexes was independently associated with presence of positive remodeling plaque.

**What Are the Clinical Implications?**

- Our study helps physicians to understand how invasive pressure measurements relate to plaque burden and aids guiding treatment decision‐making during invasive coronary angiography.

Intracoronary invasive pressure measurements have become the cornerstone to assess lesion‐specific ischemia during invasive coronary angiography (ICA).^1^, ^2^ Multiple invasive pressure indexes coexist, including resting distal pressure/arterial pressure (Pd/Pa), instantaneous wave‐free ratio (iFR), resting full‐cycle ratio (RFR), and fractional flow reserve (FFR). The most widely used index, FFR, demonstrated superior event‐free survival of an FFR‐guided revascularization strategy compared with an angiography‐guided strategy.^3^ As FFR requires a hyperemic state, RFR, iFR, and resting Pd/Pa were introduced as nonhyperemic alternatives to FFR. Among these nonhyperemic indexes, only the safety of an iFR‐guided revascularization strategy has been studied in 2 large randomized clinical trials.^4^, ^5^ Although the initial study results suggested noninferiority of iFR in comparison to FFR, pooling of data at 5‐year follow‐up revealed that iFR‐guided revascularization is associated with increased all‐cause mortality.^6^ An emerging body of evidence has shown that plaque burden and phenotype are related to outcome.^7^, ^8^, ^9^, ^10^, ^11^ Coronary plaque can be detected and characterized noninvasively by computed coronary tomography angiography (CCTA).^12^ In search of a potential explanation for the potential superiority of an FFR‐guided revascularization strategy, we previously conducted a PACIFIC1 (Comparison of Coronary CT [Computed Tomography] Angiography, SPECT [Single‐Photon Emission CT], PET [Positron Emission Tomography], and Hybrid Imaging for Diagnosis of Ischemic Heart Disease Determined by Fractional Flow Reserve) study subanalysis. Our findings revealed that FFR, when compared with iFR, exhibited greater sensitivity in detecting high‐risk plaque.^13^ In this modestly sized study, coronary plaque was quantified and phenotyped manually. We now further explored the relationship between invasive pressure indexes and atherosclerotic disease. This was endeavored by quantifying and phenotyping coronary plaque using artificial intelligence–guided quantitative coronary computed tomography angiography analysis (AI‐QCT), and by incorporating an additional prospective study cohort.^14^, ^15^, ^16^ As such, the aim of the present study was to examine the relationship between hyperemic and nonhyperemic invasive pressure indexes and both plaque burden as well as adverse plaque phenotype.

## METHODS

### Patient Population

The data that support the findings of this study are available from the corresponding author on reasonable request. This concerns a substudy of the PACIFIC1 study and ICA‐PI (Registry of Patients That Have Undergone ICA or PCI [Percutaneous Coronary Intervention], NCT04815928).^17^ The PACIFIC1 study was a prospective clinical single‐center, head‐to‐head comparative study conducted from 2012 to 2014, at the Amsterdam UMC, VU University Medical Center, Amsterdam, the Netherlands. The PACIFIC1 study included 208 patients without prior coronary artery disease who underwent CCTA before clinically indicated ICA with routine 3‐vessel pressure measurements. We included PACIFIC1 study patients with combined hyperemic and nonhyperemic pressure measurements within 1 vessel. The ICA‐PI represents a clinical cohort in which all patients who underwent ICA on clinical indication at the Amsterdam UMC were enrolled. Of patients enrolled in the ICA‐PI, all patients with a CCTA before ICA were prospectively enrolled in the CT registry. In these patients, routine combined hyperemic and nonhyperemic measurements were performed between 2020 and 2023. These patients were included for analysis. The study complied with the Declaration of Helsinki. The local ethics committee approved the PACIFIC1 study and ICA‐PI study protocols. All patients provided written informed consent.

### Computed Coronary Tomography Angiography

CT scans were performed using a 256‐slice CT scanner (Philips Brilliance iCT; Philips Healthcare, Best, the Netherlands) or a third‐generation 192‐slice dual‐source CT scanner (Somatom Force; Siemens Healthcare, Forchheim, Germany). Respective CT parameters for the 256‐ and 192‐slice dual‐source scanner entailed a section collimation of 128×0.625 mm and 192×0.600 mm and a gantry rotation time of 270 milliseconds and 250 milliseconds. Each patient received 800 μg of sublingual nitroglycerine immediately before CCTA. If necessary, metoprolol was given before CCTA by oral or intravenous administration. For visualization of the coronary artery lumen, a bolus of iobitidol (Xenetix 350) was injected intravenously, followed immediately by a saline chaser. PACIFIC1 study patients were scanned on the 256‐slice CT scanner with a tube current between 200 and 360 mA at 120 kV. Patients from the ICA‐PI were scanned on the 256‐slice or 192‐dual source CT scanner with a tube voltage between 80 and 120 Kv and an automatic tube current. Prospective ECG gating between 72% and 78% of the R‐R interval was performed to reduce radiation dose.

### AI‐Guided Quantitative CCTA Analysis

A fully automatic US Food and Drug Administration–approved AI‐based software approach (Cleerly Inc) was used to analyze the CCTA.^15^ The AI‐QCT software uses validated convolutional neural networks for image quality assessment, coronary segmentation, vessel contour determination, lumen wall evaluation, and plaque characterization and quantification. First, the algorithm produces a centerline, lumen, and outer vessel wall contours for every phase available. Subsequently, the algorithm selects the 2 optimal series for analysis for each coronary artery. Following automated segmentation and labeling of all coronary arteries, plaques are characterized and quantified on the basis of Hounsfield unit attenuation. Finally, the AI‐determined center line and vessel wall contours are supervised by a radiologic technologist for quality assurance review and, if necessary, adjusted. Coronary segments with a diameter ≥1.5 mm were included for analysis. AI‐QCT analyses were performed on a per‐segment basis using the modified 18‐segment Society of Cardiovascular Computed Tomography model.^18^ Segments were evaluated for the presence of coronary atherosclerosis, defined as any tissue structure >1 mm^2^ within the coronary artery wall that was differentiated from the surrounding epicardial tissue, epicardial fat, or the vessel lumen itself. Plaque volumes (mm^3^) were calculated for each coronary lesion and then summated to compute the total plaque volume for the entire segment. Vessel‐specific plaque volume was normalized to the vessel volume to account for variation in coronary artery volume, calculated as vessel‐specific plaque volume/vessel‐specific volume×100%. These normalized volumes were reported as percentage atheroma volume (PAV). Within coronary artery lesions, plaque was characterized as low‐attenuation plaque (LAP), noncalcified plaque, and calcified plaque based on Hounsfield units of <30, 30 to 350, >350.^19^ LAP was defined as plaques with any component <30 Hounsfield units on a pixel‐level basis. Positive arterial remodeling (PR) was identified as a remodeling index >1.1 by diameter in comparison to a proximal vessel reference.^19^ High‐risk plaque was considered plaque with both a positive remodeling index >1.1 and LAP. Plaque and vessel volumes were summed from the position of the aortic pressure sensor until the distal pressure sensor. If image quality was insufficient or artifacts hindered analysis, only the slices with insufficient quality were excluded from analysis. The AI‐QCT algorithm has been validated against expert CT readers, quantitative coronary angiography, and intravascular ultrasound.^14^, ^15^, ^20^, ^21^ Furthermore, AI‐QCT–derived quantitative plaque analysis showed important prognostic value in addition to clinical risk factors, coronary calcium score, diameter stenosis (DS%), and Coronary Artery Disease‐Reporting and Data System.^11^, ^16^ The radiologic technologists who performed quality assurance review were blinded to the results of the invasive coronary pressure measurements.

### Invasive Coronary Angiography

ICA was performed according to standard clinical protocols.^17^ A 0.014‐inch sensor‐tipped guidewire, introduced through a 5F or 6F guiding catheter, was used to measure pressure. In the PACIFIC1 study, routine 3‐vessel pressure measurements were performed. Operators refrained from pressure measurement if they were considered unsafe or technically unfeasible. First, nonhyperemic coronary pressure measurements were performed while care was taken to avoid preceding intracoronary saline or contrast injection. The nonhyperemic measurements were followed by induction of maximal coronary hyperemia through administration of adenosine either systemically through intravenous infusion of 140 μg/kg per minute into a large vein, or intracoronary as a 150 μg bolus. At the end of the procedure, pressure drift was assessed and in case of >2 mm Hg drift, the measurement was either repeated or pressure drift was corrected for in the final analysis. All pressure indexes were calculated as the ratio between distal pressure and aortic pressure. FFR and Pd/Pa were defined as the mean over the entire cardiac cycle.^22^, ^23^ iFR was measured during the wave‐free period.^22^ RFR was defined as the minimal value of Pd/Pa.^22^ RFR, iFR, and Pd/Pa were assessed under resting conditions, whereas FFR was obtained during adenosine‐induced hyperemic conditions.^22^ Previously designated cutoff values for hemodynamic significance were as follows: iFR/RFR ≤0.89, Pd/Pa ≤0.92, and FFR ≤0.80.^3^, ^24^, ^25^ In the PACIFIC1 study, Pd/Pa and iFR were obtained as nonhyperemic indexes, whereas in the CT registry, Pd/Pa and RFR were obtained. The VALIDATE RFR study demonstrated that RFR and iFR were diagnostically equivalent (R^2^=0.99, *P*<0.001).^24^ As such, to assess the association with plaque volumes for combined data of the PACIFIC1 study and CT registry, the included resting indexes were Pd/Pa and either RFR or iFR. FFR was measured in both patient cohorts. Each coronary artery was visually assessed for luminal narrowing using at least 2 orthogonal views and scored as no stenosis (0% DS), 1% to 29% DS, 30% to 49% DS, 50% to 69% DS, 70% to 99% DS, or total occlusion (100% DS).

### Statistical Analysis

Continuous variables are expressed as mean±SD or median (interquartile range), where appropriate. Categorical variables are presented as frequencies with percentages. Normality of data was examined by means of QQ plots and histograms. Baseline characteristics between the 2 cohorts were compared by the independent sample *t* test for continuous variables and the χ^2^ test for categorical variables. Invasive pressure measurements were matched with vessel‐specific plaque volumes and characteristics. The correlation between invasive pressure indexes and PAV was assessed by Spearman correlation coefficient. Spearman correlations between the different invasive pressure indexes and plaque volumes were compared using z‐tests on Fisher z‐transformed correlation coefficients. Levels of PAV stratified according to invasive pressure measurements results were compared by the Kruskal‐Wallis test. In case the overall Kruskal‐Wallis test was significant, post hoc pairwise comparison between vessel categories were performed by the Mann‐Whitney *U* test with Bonferroni correction for multiple comparisons. To adjust for multiple vessels within a single subject, mixed models with a random intercept at the subject level were used. These models were used to evaluate the adjusted associations between the independent variables (PAV, PR, LAP, and ICA‐determined DS%) and the invasive pressure indexes as the dependent variable. Additional mixed models were constructed with PR as a continuous variable instead of using the >1.1 cutoff and high‐risk plaque (plaque with both PR and LAP) instead of the individual plaque characteristics. Moreover, invasive pressure measurement results were stratified according to the PAV stages, as proposed by Min et al.^26^ Although these stages are validated as a per‐patient parameter, PAV is corrected for vessel volume so it can be used as a per‐vessel parameter as well. A 2‐sided *P*<0.05 was considered statistically significant. All statistical analyses were performed using IBM SPSS software package, version 28 (IBM SPSS Statistics, IBM Corporation, Armonk, NY).

## RESULTS

**Figure 1:**
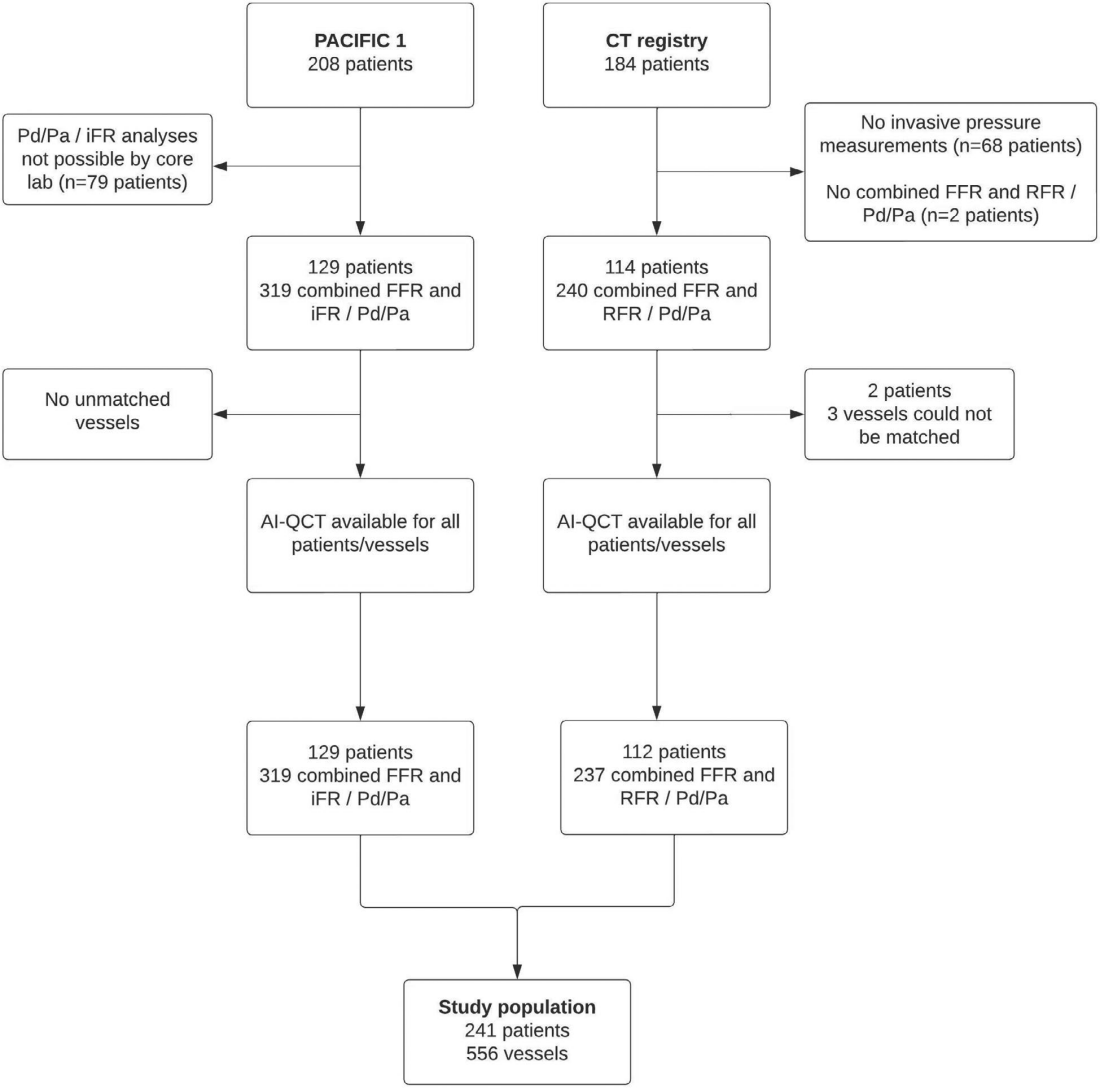
Patient inclusion flowchart. AI‐QCT indicates artificial intelligence–guide quantitative coronary computed tomography angiography; CT, computed tomography; FFR, fractional flow reserve; iFR, instantaneous wave‐free ratio; PACIFIC1, Comparison of Coronary CT Angiography, SPECT (Single‐Photon Emission CT), PET (Positron Emission Tomography), and Hybrid Imaging for Diagnosis of Ischemic Heart Disease Determined by Fractional Flow Reserve; Pd/Pa, resting distal pressure/arterial pressure; and RFR, resting full‐cycle ratio.

In total, the study population consisted of 241 patients in whom 556 vessels were interrogated by both resting and hyperemic intracoronary pressure measurements as well as vessel‐specific AI‐QCT plaque analysis (Figure 1). Patients originated from the PACIFIC1 study (129 patients, 319 vessels) and the CT registry (112 patients, 237 vessels). The mean age was 61±9 years, and 168 (70%) were men. Baseline characteristics were largely similar between patients from the PACIFIC1 study cohort and the CT registry. Patients from the CT registry, however, were older (64±9 versus 58±9 years; *P*<0.01; Table 1). Vessel characteristics are described in Table 2. Adenosine was administered as an intracoronary bolus in 466 (84%) FFR interrogations.

**Table 1:** Baseline Characteristics

Characteristic	All (n=241)	PACIFIC1 study (n=129)	CT registry (n=112)	*P* value
Male sex	168 (70)	83 (64)	85 (76)	0.05
Age, y	60.9±9.4	58.3±8.6	63.9±9.4	<0.01
BMI, kg/m^2^	26.5±3.7	26.6±3.6	26.2±3.8	0.26
Cardiovascular risk factors				
Diabetes	35 (15)	20 (16)	15 (14)	0.66
Hypertension	111 (46)	60 (47)	51 (46)	0.98
Hypercholesterolemia	97 (40)	48 (37)	49 (44)	0.25
Current smoker	41 (17)	28 (22)	13 (12)	0.10
Family history of CAD (n=220)	118 (49)	66 (51)	52 (46)	0.38
Medication				
Antiplatelet therapy	200 (41)	111 (86)	89 (80)	0.18
β‐Blocker	152 (63)	85 (66)	67 (60)	0.33
Calcium channel blocker	66 (27)	37 (29)	29 (26)	0.63
ACE inhibitor	41 (17)	24 (19)	17 (15)	0.48
ARB	44 (18)	26 (20)	18 (16)	0.41
Statin	179 (74)	95 (74)	84 (75)	0.81
Symptoms				
Typical angina	86 (36)	48 (37)	38 (34)	0.60
Atypical angina	57 (24)	34 (26)	23 (21)	0.29
Nonspecific chest discomfort	31 (13)	24 (19)	7 (6)	<0.01
Other	67 (27)	23 (18)	44 (39)	<0.01

Data are given as number (percentage) or mean±SD. ACE indicates angiotensin‐converting‐enzyme; ARB, angiotensin II receptor blocker; BMI, body mass index; CAD, coronary artery disease; CT, computed tomography; and PACIFIC1, Comparison of Coronary CT Angiography, SPECT (Single‐Photon Emission CT), PET (Positron Emission Tomography), and Hybrid Imaging for Diagnosis of Ischemic Heart Disease Determined by Fractional Flow Reserve.

**Table 2:** Per‐Vessel Angiographic and CT Findings

Findings	All vessels (n=556)
Included vessel	
Left anterior descending artery	193 (35)
Left circumflex artery	137 (25)
Right coronary artery	156 (28)
Side branch	70 (13)
Invasive angiographic characteristics	
No stenosis	242 (44)
1%–29%	27 (5)
30%–49%	102 (18)
50%–69%	92 (17)
70%–99%	82 (15)
Pd/Pa	0.98 (0.93–1.00)
iFR/RFR	0.96 (0.90–1.00)
FFR	0.93 (0.84–0.98)
CT plaque characteristics	
% Atheroma volume (1.00 indicates 100%)	0.11 (0.03–0.24)
Presence of positive remodeling plaque	442 (76)
Presence of low‐attenuation plaque	165 (30)

Data are given as number (percentage) or median (interquartile range). CT indicates computed tomography; FFR, fractional flow reserve; iFR, instantaneous wave‐free ratio; Pd/Pa, resting distal pressure/arterial pressure; and RFR, resting full‐cycle ratio.

### Relation of Invasive Pressure Measurements to PAV

**Figure 2:**
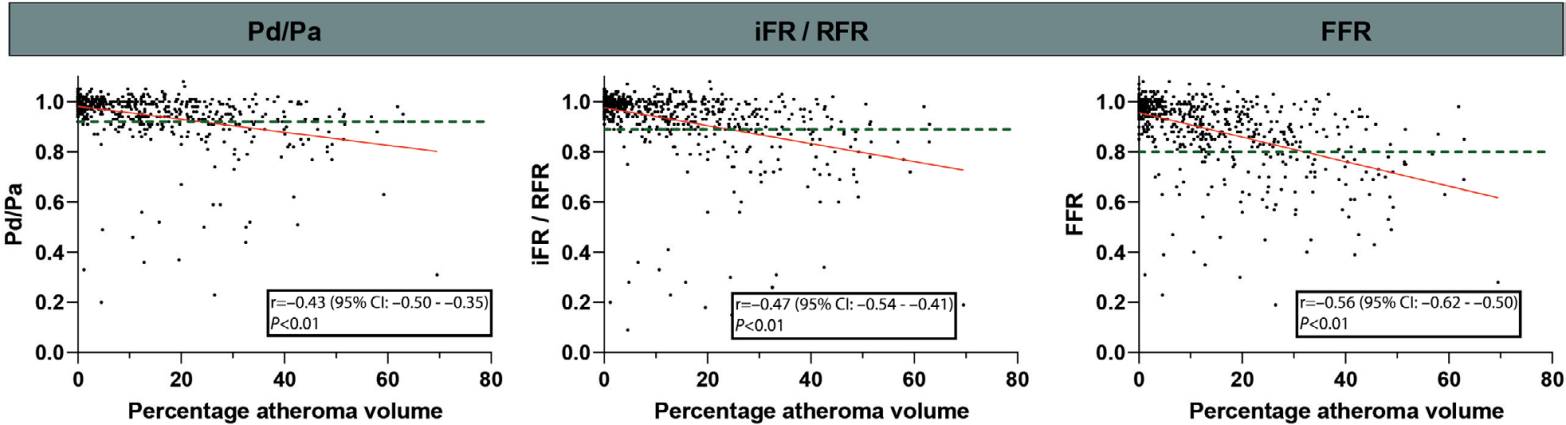
The correlation between invasive pressure indexes and PAV. The correlation between invasive pressure measurements and PAV. The dotted green line indicates the cutoff for normal or abnormal measurements, respectively. FFR indicates fractional flow reserve; iFR, instantaneous wave‐free ratio; PAV, percentage atheroma volume; Pd/Pa, resting distal pressure/arterial pressure; and RFR, resting full‐cycle ratio.

**Figure 3:**
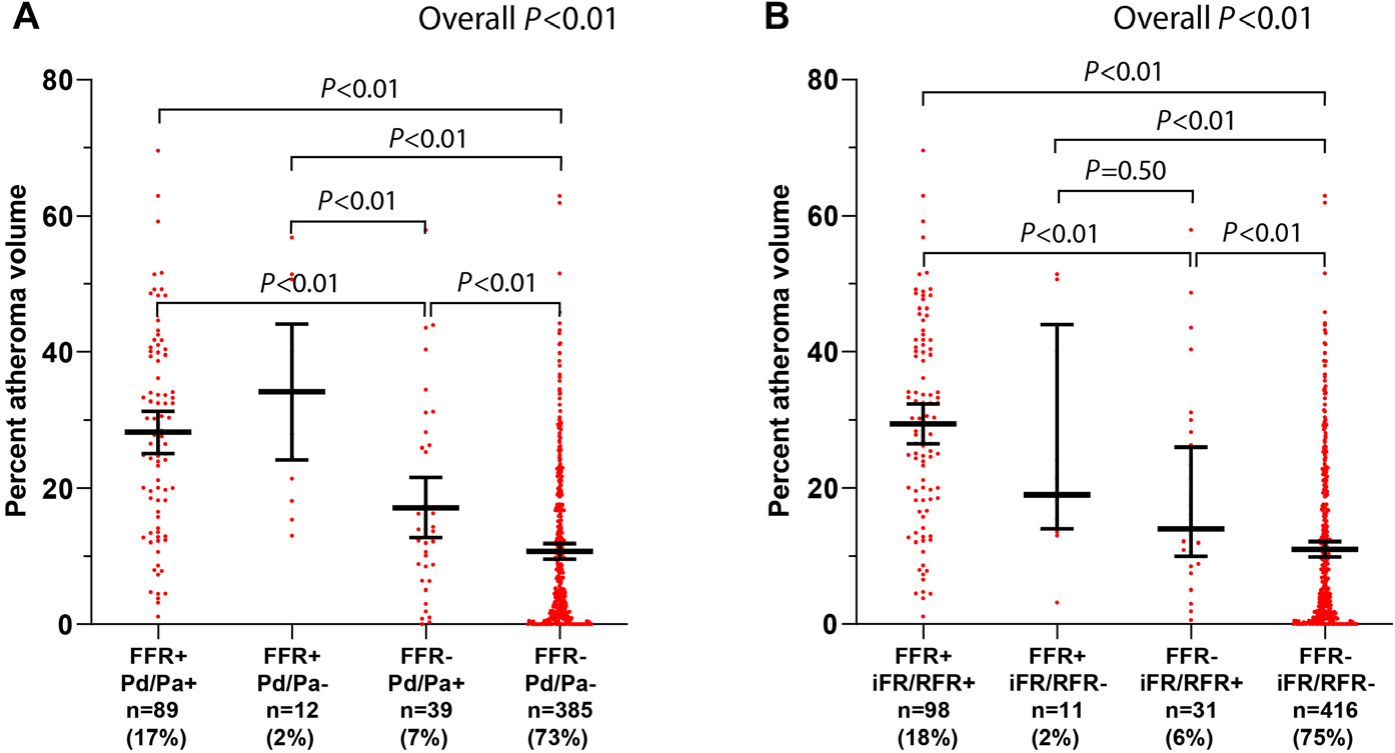
PAV according to combined invasive pressure measurements. Median levels (95% CI) of PAV stratified according to invasive pressure measurement results. **A**, Combined FFR and Pd/Pa results are shown. **B**, Combined FFR and iFR/RFR results are shown. FFR, fractional flow reserve; iFR, instantaneous wave‐free ratio; PAV, percentage atheroma volume; Pd/Pa, resting distal pressure/arterial pressure; RFR, resting full‐cycle ratio.

Fair to moderate correlations were observed between different pressure indexes and PAV (Figure 2). The correlation between FFR and PAV was stronger than the correlation between Pd/Pa and PAV (r=−0.56 [95% CI, −0.62 to −0.50] versus r=−0.43 [95% CI, −0.50 to −0.35]; *P*<0.01). In accordance, the correlation between FFR and PAV was also stronger than between iFR/RFR and PAV (r=−0.47 [95% CI, −0.54 to −0.41]; *P*=0.04 for difference). Similar findings were observed after stratification on patient cohort (Table S1). The correlation between PAV and invasive pressure indexes in intermediate coronary stenoses (30%–70% DS) was more modest, but with similar patterns (Pd/Pa r=−0.33 [95% CI, −0.46 to −0.19]; iFR/RFR r=−0.32 [95% CI, −0.44 to −0.18]; FFR r=−0.40 [95% CI, −0.52 to −0.28]; *P*<0.01 for all). Figure 3 depicts the amount of PAV stratified according to invasive measurement results. It can be appreciated from Figure 3A that FFR+ Pd/Pa− vessels showed higher PAV than FFR− Pd/Pa+ vessels (34% [95% CI, 19%–50%] versus 14% [95% CI, 8%–26%]; *P*<0.01). A similar trend was observed for vessels stratified on FFR and iFR/RFR (Figure 3B). However, FFR+ iFR/RFR− and FFR− iFR/RFR+ did not differ significantly (21% [95% CI, 14%–44%] versus 14% [95% CI, 10%–26%]; *P*=0.50 after Bonferroni correction). Similar patterns were observed for noncalcified plaque volume stratified on invasive pressure measurement results. All pressure indexes were associated with PAV in a univariable analysis (Table 3). In a multivariable analysis, incorporating DS%, PAV, LAP, and PR, FFR was associated with PAV (β, −0.15, *P*<0.01), whereas PAV was not independently associated with Pd/Pa (β, −0.06, *P*=0.12) or iFR/RFR (β, −0.08, *P*=0.08; Table 3). This is also illustrated by Figure S2, which depicts the median (95% CI) for the different pressure indexes stratified for plaque stages according to Min et al.^26^ It is notable that stages 0 and 1 show overlapping 95% CIs for FFR values, whereas stages 2 and 3 have distinctly nonoverlapping 95% CIs. In contrast, the 95% CIs for iFR/RFR or Pd/PA overlap for stages 0 through 2.

**Table 3:** Association Between Invasive Pressure Indexes and Plaque Quantity and Phenotype

Variable	Pd/Pa		iFR/RFR		FFR	
	β	*P* value	β	*P* value	β	*P* value
Univariable analysis						
No stenosis (reference)	…	…	…	…	…	…
1%–29%	−0.00	<0.01	−0.02	0.51	−0.03	0.14
30%–49%	−0.03	<0.01	−0.04	<0.01	−0.08	<0.01
50%–69%	−0.07	0.01	−0.09	<0.01	−0.14	<0.01
70%–99%	−0.19	0.02	−0.25	<0.01	−0.29	<0.01
PAV (per 0.01 increase)	−0.26	<0.01	−0.37	<0.01	−0.50	<0.01
Positive remodeling plaque	−0.05	<0.01	−0.07	<0.01	−0.09	<0.01
Low‐attenuation plaque	−0.07	<0.01	−0.10	<0.01	−0.12	<0.01
Multivariable analysis						
No stenosis (reference)	…	…	…	…	…	…
1%–29%	0.02	0.93	−0.01	0.72	−0.20	0.32
30%–49%	−0.01	0.25	−0.02	0.15	−0.05	<0.01
50%–69%	−0.05	<0.01	−0.07	<0.01	−0.10	<0.01
70%–99%	−0.17	<0.01	−0.22	<0.01	−0.25	<0.01
PAV (per 0.01 increase)	−0.06	0.12	−0.08	0.08	−0.15	<0.01
Positive remodeling plaque	−0.01	0.57	−0.01	0.72	−0.01	0.36
Low‐attenuation plaque	−0.02	0.10	−0.03	0.01	−0.03	<0.01

FFR indicates fractional flow reserve; iFR, instantaneous wave‐free ratio; PAV, percentage atheroma volume; Pd/Pa, resting distal pressure/arterial pressure; and RFR, resting full‐cycle ratio.

### Relation of Invasive Pressure Measurement to Adverse Plaque Phenotype

**Figure 4:**
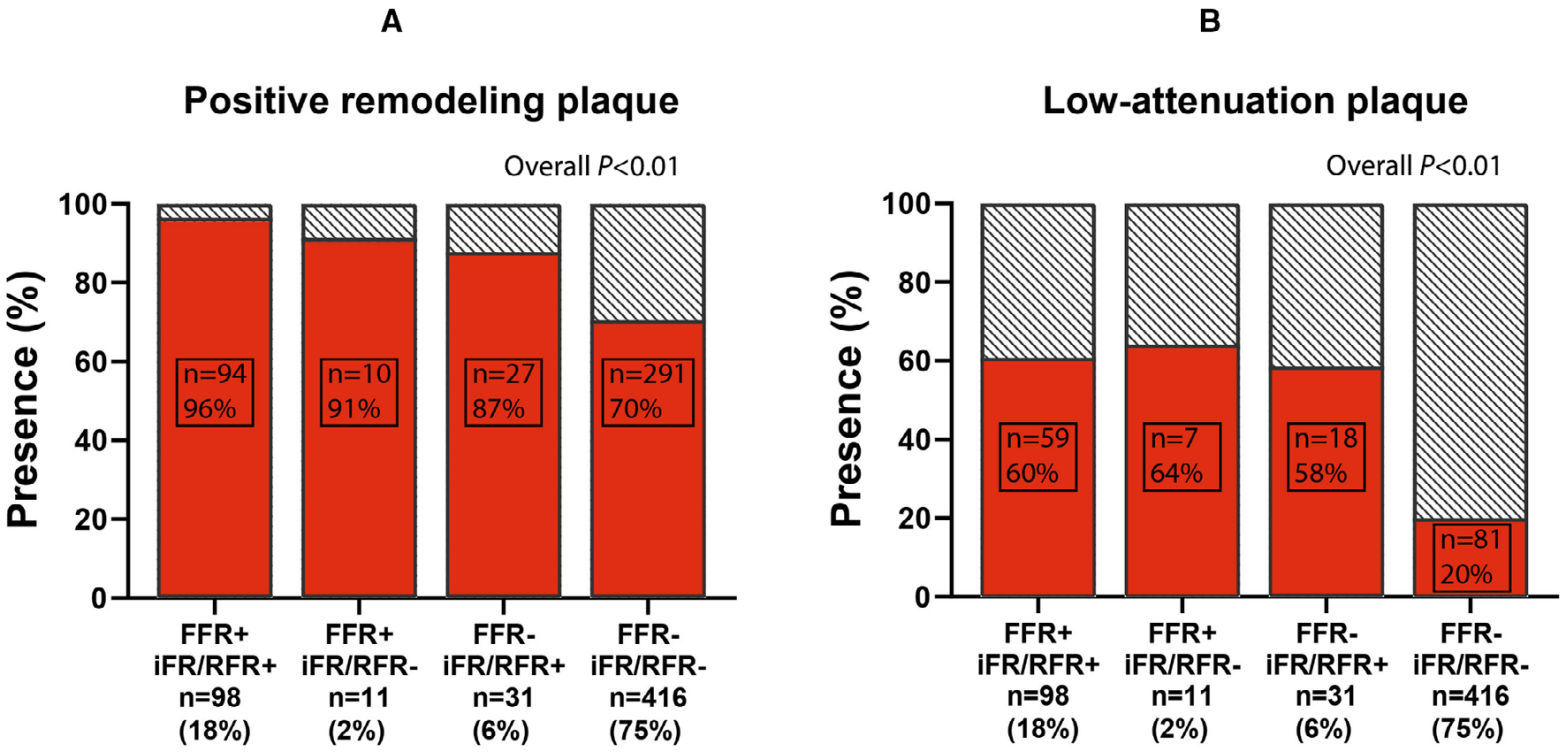
Adverse plaque characteristics according to FFR and iFR/RFR results. **A**, Combined FFR and Pd/Pa results are shown. **B**, Combined FFR and iFR/RFR results are shown. FFR indicates fractional flow reserve; iFR, instantaneous wave‐free ratio; Pd/Pa, resting distal pressure/arterial pressure; and RFR, resting full‐cycle ratio.

Figure 4 and Figures S3 and S4 depict the number of vessels with PR or LAP, stratified according to FFR, iFR/RFR, and Pd/Pa results. In general, plaques with PR occurred frequently in all interrogated vessels (n=442, 76% of all vessels; Table 2). PR plaques were more present in FFR+ iFR/RFR+ than FFR− iFR/RFR− vessels (96% versus 70%; *P*<0.01), but did not occur more often in FFR+ iFR/RFR− vessels than in FFR− iFR/RFR+ vessels (91% versus 87%; *P*=0.66). LAP was present in 165 vessels (30%). LAP was more frequently observed in FFR+ iFR/RFR+ vessels than FFR− iFR/RFR− vessels (60% versus 20%), but equally distributed among FFR+ iFR/RFR− and FFR− iFR/RFR+ vessels (64% versus 58%; *P*=0.87). Similar results were observed after stratification for FFR and Pd/Pa (Figure S3). None of the invasive pressure measurements was independently significantly associated with PR in a multivariable analysis (Table 3). PR as a continuous variable was not independently associated with any of the invasive pressure indexes as well (*P*>0.23 for all). FFR and iFR/RFR, but not Pd/Pa, were independently significantly associated with the presence of LAP (β, −0.03, *P*<0.01; β, −0.03, *P*=0.01; and β, −0.02, *P*=0.10). Similar results were found after stratification for patient cohort (Table S2). FFR and iFR/RFR were associated with high‐risk plaque (β, −0.02, *P*=0.04 for FFR and β, −0.03, *P*<0.01 for iFR/RFR), whereas Pd/Pa was not (β, −0.02, *P*=0.14; Table S3).

## DISCUSSION

In this post hoc study of prospectively collected data using a reproducible, operator‐independent AI tool to assess plaque quantity and phenotype as well as invasive pressure measurements, the main findings could be summarized as follows: FFR correlated stronger to PAV compared with nonhyperemic pressure indexes; both FFR and iFR/RFR were independently associated with the presence of LAP, but Pd/Pa was not; none of the invasive pressure indexes was independently associated with the presence of PR plaque.

The relationship between atherosclerotic disease and invasive pressure measurements has gained additional attention since a pooled analysis of the long‐term safety studies of iFR has shown adverse results in iFR‐guided treated patients in comparison to FFR‐guided treated patients.^6^ These outcome differences are remarkable because discordance exists only in 20% of interrogated vessels.^24^ Multiple theories have been proposed to explain the potential iFR inferiority. One plausible explanation is that discordant results have been more frequently reported in left main or proximal left anterior descending coronary artery lesions, where plaques have been associated with a worse prognosis.^27^, ^28^, ^29^ Moreover, we found in a prior study that FFR related stronger to adverse plaque characteristics compared with iFR.^13^

Historically, the focus of CCTA was to detect coronary stenosis; however, the extent of coronary atherosclerosis might better capture a patient's risk for future events.^30^ In that light, the current study contributes to the accumulated evidence indicating that FFR provides a more comprehensive assessment of total plaque burden than vasodilator‐free indexes, like iFR, RFR, and Pd/Pa. By identifying not only high‐grade stenoses but also the total plaque burden of vessels, FFR might aid in the appropriate classification of vessels and more precisely guide coronary revascularization and medical therapy. Furthermore, the observation that most acute coronary syndrome culprit lesions were nonobstructive encouraged the identification of rupture‐prone plaque on CCTA.^10^ This led to the identification of the adverse plaque phenotype as it has been associated with adverse outcome.^31^ Total plaque burden demonstrated a greater prognostic power than the number of plaques with PR or LAP volume.^11^ Several smaller CCTA studies have reported that FFR in comparison to iFR was superiorly associated with adverse plaque characteristics.^13^, ^32^, ^33^ In contrast, we found that both FFR and iFR/RFR, but not Pd/Pa, associated with the presence of LAP, independent of stenosis grade and plaque burden. Moreover, none of the invasive pressure indexes was independently associated with the presence of PR plaque. Of the above‐mentioned studies, Lee et al studied the correlation between invasive pressure indexes and adverse plaque characteristics but did not correct for total plaque volume. Therefore, these results could partially be explained by the superior potential of FFR to detect plaque burden rather than its relationship with adverse plaque characteristics in specific. A second explanation could be in the method of defining adverse plaque characteristics. Four adverse plaque characteristics are usually reported: LAP, PR, spotty calcifications, and the napkin ring sign.^13^ Our AI‐QCT analysis tool only reports LAP and PR, preluding the assessment of spotty calcifications and the napkin ring sign. Also, plaque characterization in the study of Driessen et al was meticulously performed by a dedicated core laboratory, which might detect subtle differences that are lost in AI‐QCT analyses, as illustrated by only a fair κ between AI‐QCT and manual‐level 3 CT readers to detect high‐risk plaque.^21^ Importantly, the interobserver variability to phenotype adverse plaque characteristics among CCTA expert readers has been reported to be only fair to moderate too.^34^ This strongly illustrates the necessity of validated and reproducible measures of atherosclerotic imaging.

The wide scatter and modest correlation between the invasive pressure indexes and PAV is not unexpected. Although FFR reflects the functional significance of coronary stenoses, PAV serves as an anatomic measure. For instance, PAV concentrated over a short segment can lead to a high‐grade stenosis and a low FFR. In contrast, the same amount of PAV distributed over a longer vessel segment may indicate nonobstructive coronary artery disease with a relatively preserved FFR. Therefore, these indexes are not interchangeable.^35^ FFR has been extensively validated as a tool for guiding treatment decision‐making during ICA, whereas CCTA‐derived measures of plaque burden and adverse characteristics have primarily been used to assess an individual's risk of future events and guide medical therapy.^3^, ^11^, ^30^, ^36^ The application of these CCTA‐derived indexes for ICA decision‐making has been more limited. Ideally, invasive treatment of coronary plaque would address both functional significance and high‐risk plaque features. However, the integration of these functional and anatomic indexes during ICA remains relatively underexplored. Yang et al provided valuable insight into this interplay, showing that combining low FFR (≤0.70) with a high pullback pressure gradient (>0.65) could identify vulnerable plaques, with 88% of vessels exhibiting low FFR and high pullback pressure gradient demonstrating adverse plaque features.^37^ Our study expands on this work by comparing hyperemic and nonhyperemic indexes to further explore these relationships.

A fundamental difference between hyperemic and nonhyperemic pressure measurements is the influence of the microcirculation, which is obliviated after the administration of a vasodilator. Therefore, a pressure decrease across a coronary stenosis under nonhyperemic conditions is not exclusively related to the severity of the stenosis and plaque burden. It is, in fact, related to the vasodilatory capacity of the microcirculation, and not necessarily informative about the degree of reduction in coronary flow attributable to the stenosis.^22^ In that light, our data emphasize the importance of selecting the appropriate diagnostic tool for assessing the severity of coronary artery disease as it might not only guide coronary intervention but also direct noninvasive (medical) therapy.

### Limitations

This is a post hoc analysis with inherent study limitations. First, a substantial part of patients has been recently enrolled and, therefore, relevant clinical follow‐up data have yet to be collected. Second, most interrogated vessels showed concordant invasive pressure measurements, limiting the sample size to detect clinically relevant differences after binary stratification (normal versus abnormal invasive pressure measurements). Third, our AI‐QCT analysis tool did not report the presence of spotty calcifications nor the napkin ring sign. As such, we could not study the impact of these adverse plaque characteristics. Fourth, PACIFIC1 study patients were scanned using the same Kv settings, whereas an adaptive Kv approach was used in the CT registry. Although overall PAV is unlikely to be affected by difference in scanners or Kv, these parameters might have impacted plaque characterization.^38^ Fifth, patients were enrolled from 2 different cohorts, which could potentially introduce bias. However, we believe that this bias is minimal because the patients were enrolled consecutively. More importantly, all patients underwent both rest –and hyperemic invasive pressure measurements of the same vessel. As a result, it is unlikely that selection bias could have affected our findings as there is no bias in which patients or vessels have been interrogated with hyperemic or rest invasive pressure indexes. Sixth, we adjusted our analyses for stenosis percentage, which is only 1 of the many factors that influence coronary flow and coronary pressure measurements. Nevertheless, we do not expect that adjusting for additional variables would affect the results, as all measurements were taken from the same vessels with the same characteristics. Last, vessels were matched on the basis of manual designation on ICA‐ and AI‐QCT–guided nomenclature on CCTA. Although the same nomenclature was used, a small number of vessels could not be matched, and it cannot be excluded that several vessels have been matched incorrectly. However, potential mismatches are unlikely to significantly affect our findings, as any incorrectly matched vessels would be subject to all invasive pressure indexes.

## CONCLUSIONS

The present study suggests that FFR provides a superior assessment of plaque burden compared with nonhyperemic pressure indexes. Discordant vessels with abnormal FFR but normal Pd/Pa exhibited higher plaque burden than vessels with normal FFR but abnormal Pd/Pa, whereas a similar plaque burden was observed for FFR and iFR/RFR discordant vessels. Both FFR and iFR/RFR were associated with the presence of LAP, whereas Pd/Pa was not. None of the invasive pressure indexes was independently associated with the presence of PR.

## Sources of Funding

This work was internally funded.

## Disclosures

Dr Nurmohamed reports grants from the Dutch Heart Foundation (Dekker 03–007–2023‐0068), European Atherosclerosis Society (2023), research funding/speaker fees from Cleerly, Daiichi Sankyo, and Novartis, and is cofounder of Lipid Tools; Dr Danad has received a research grant from Cleerly Inc; Dr Knaapen has received research grants from HeartFlow and Cleerly, Inc. The remaining authors have no disclosures to report.

## Supporting information

Tables S1–S3
Figures S1–S4
