# Supplementary material for: Fractional Flow Reserve Relates Stronger to Coronary Plaque Burden Than Nonhyperemic Pressure Indexes
Source: J Am Heart Assoc. 2025 Feb 19;14(5):e039324. doi: 10.1161/JAHA.124.039324 (PMC12132701; doi:10.1161/JAHA.124.039324)
Supplement: Supplementary file 1 — Tables S1–S3 Figures S1–S4 [file JAH3-14-e039324-s001.pdf]

SUPPLEMENTAL MATERIAL

Table S1. Correlation between PAV and invasive pressure indices stratified according to patient cohort.

	PAV			
	**PACIFIC1**	**P-value**	**CT registry**	**P-value**
**Pd/Pa**	-0.39 (-0.48 - -0.29)	<0.01	-0.43 (-0.54 - -0.31)	<0.01
**iFR/RFR**	-0.43 (-0.52 - -0.33)	<0.01	-0.43 (-0.54 - -0.31)	<0.01
**FFR**	-0.58 (-0.65 - -0.50)	<0.01	-0.49 (-0.58 - -0.38)	<0.01

Abbreviations: Abbreviations: FFR, fractional flow reserve; iFR, instantaneous wave-free ratio; PAV, percentage atheroma volume; Pd/Pa, resting distal pressure/arterial pressure; RFR, resting full-cycle ratio

Table S2. The independent association between invasive pressure indices and plaque quantity and phenotype stratified according to patient cohort

	Pd/Pa	P-value	iFR/RFR	P-value	FFR	P-value
	Beta	P-value	Beta	P-value	Beta	P-value
PACIFIC 1						
No stenosis (ref)	-	-	-	-	-	-
1%-29%	0.01	0.74	0.02	0.75	-0.00	0.98
30%-49%	-0.01	0.55	-0.02	0.38	-0.06	<0.01
50%-69%	-0.07	<0.01	-0.10	<0.01	-0.14	<0.01
70%-99%	-0.27	<0.01	-0.36	<0.01	-0.34	<0.01
PAV	-0.08	0.16	-0.14	0.05	-0.20	<0.01
Positive remodeling	-0.01	0.51	-0.01	0.68	-0.10	0.48
Low-attenuation	-0.01	0.71	-0.01	0.73	-0.01	0.58
CT registry						
No stenosis (ref)	-	-	-	-	-	-
1%-29%	-0.00	0.84	-0.01	0.60	-0.03	0.15
30%-49%	-0.02	0.22	-0.02	0.21	-0.05	<0.01
50%-69%	-0.02	0.31	-0.02	0.28	-0.06	<0.01
70%-99%	-0.13	<0.01	-0.16	<0.01	-0.23	<0.01
PAV	-0.05	0.30	-0.06	0.28	-0.09	0.09
Positive remodeling	-0.02	0.35	-0.01	0.45	-0.03	0.13
Low-attenuation	-0.01	0.25	-0.03	0.04	-0.03	0.04

Regression coefficients from multivariable linear regression models with Pd/Pa, iFR/RFR and FFR as dependent variables

Abbreviations: as in Table S1

Table S3. The independent relation between invasive pressure indices and high-risk plaque

Multivariable analysis						
	**Pd/Pa**		**iFR/RFR**		**FFR**	
	Beta	P-value	Beta	P-value	Beta	P-value
No stenosis (ref)	-	-	-	-	-	-
1%-29%	0.00	0.91	-0.01	0.70	-0.02	0.35
30%-49%	-0.02	0.18	-0.02	0.10	-0.06	<0.01
50%-69%	-0.05	<0.01	-0.07	<001	-0.11	<0.01
70%-99%	-0.17	<0.01	-0.22	<0.01	-0.25	<0.01
PAV (per 0.01 increase)	-0.07	0.05	-0.09	0.05	-0.17	<0.01
High-risk plaque	-0.02	0.14	-0.03	<0.01	-0.02	0.04

Figure S1. Non-calcified plaque according to invasive pressure measurements

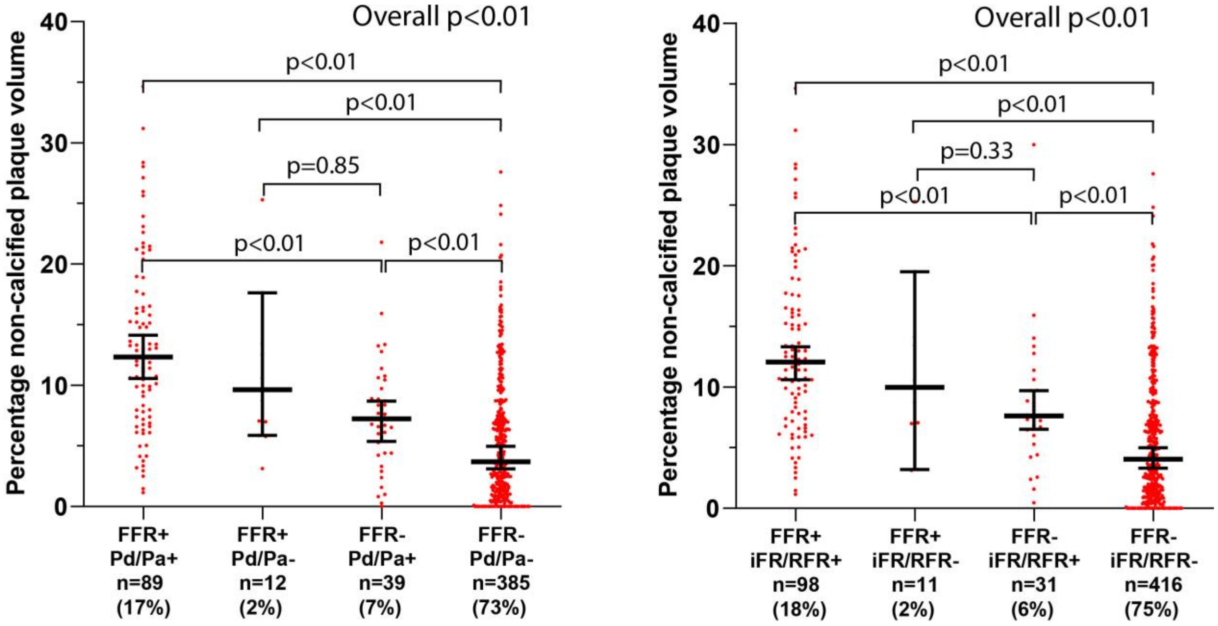

Median invasive pressure measurement results with 95% CIs. Abbreviations: as in Table S1

Figure S2. PAV according to plaque stages

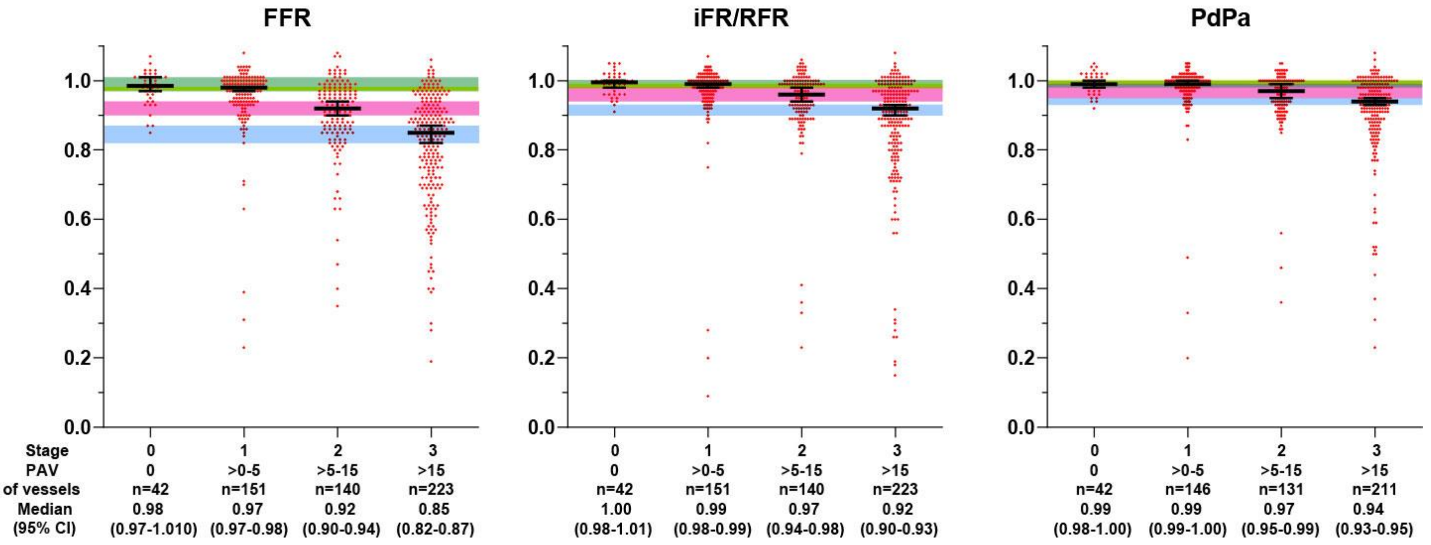

Median invasive pressure measurement results with their 95% CIs stratified according to PAV stages. The 95% CIs per plaque stage are colored and depicted across all plaque stages to illustrate their overlap.

Abbreviations: as in Table S1

Figure S3. Adverse plaque characteristics in relation to FFR and Pd/Pa results

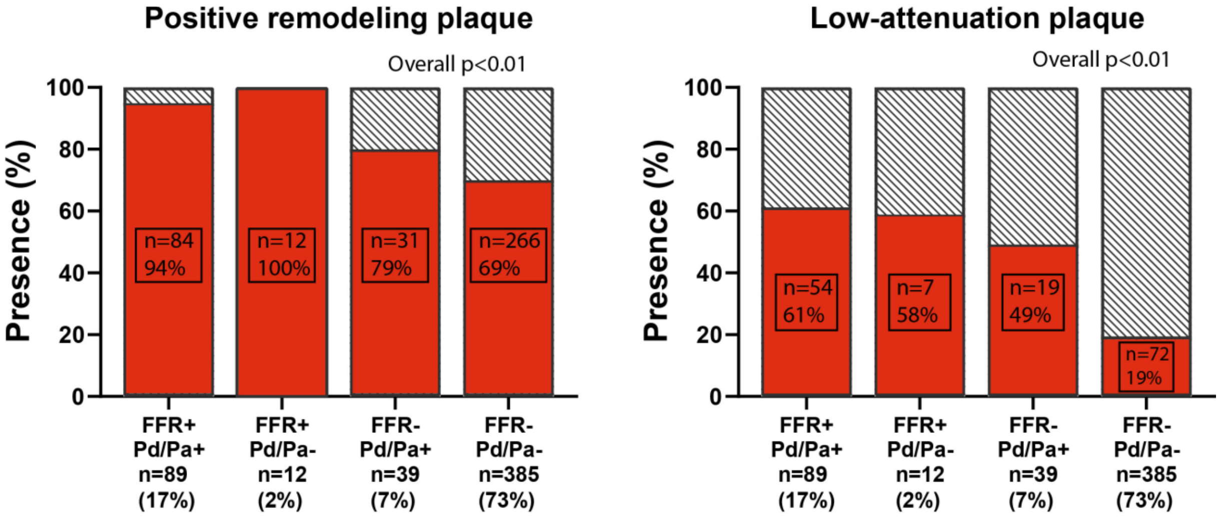

Abbreviations: as in Table S1

Figure S4. Vessels with 2 adverse plaque characteristics in relation to invasive pressure measurements

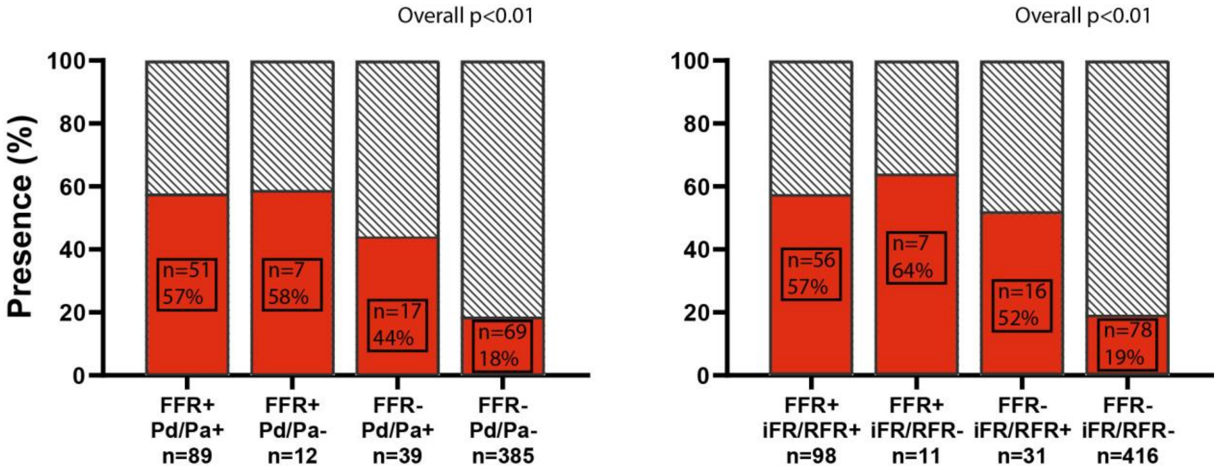

Abbreviations: as in Table S1
